# Global Autorecognition and Activation of Complement by Mannan-Binding Lectin in a Mouse Model of Type 1 Diabetes

**DOI:** 10.1155/2017/9403754

**Published:** 2017-06-13

**Authors:** Esben Axelgaard, Jakob Appel Østergaard, Saranda Haxha, Steffen Thiel, Troels Krarup Hansen

**Affiliations:** ^1^Department of Biomedicine, Wilhelm Meyer's Allé 4, Faculty of Health Sciences, Aarhus University, Aarhus C, Denmark; ^2^Department of Endocrinology and Internal Medicine, Aarhus University Hospital, Aarhus, Denmark; ^3^The Danish Diabetes Academy, Odense, Denmark

## Abstract

Increasing evidence links mannan-binding lectin (MBL) to late vascular complications of diabetes. MBL is a complement-activating pattern recognition molecule of the innate immune system that can mediate an inflammation response through activation of the lectin pathway. In two recent animal studies, we have shown that autoreactivity of MBL is increased in the kidney in diabetic nephropathy. We hypothesize that long-term exposure to uncontrolled high blood glucose in diabetes may mediate formation of neoepitopes in several tissues and that MBL is able to recognize these structures and thus activate the lectin pathway. To test this hypothesis, we induced diabetes by injection of low-dose streptozotocin in MBL double-knockout (MBL/DKO) mice. Development of diabetes was followed by measurements of blood glucose and urine albumin-to-creatinine ratio. Fluorophore-labelled recombinant MBL was injected intravenously in diabetic and nondiabetic mice followed by ex vivo imaging of several organs. We observed that MBL accumulated in the heart, liver, brain, lung, pancreas, and intestines of diabetic mice. We furthermore detected increased systemic complement activation after administration of MBL, thus indicating MBL-mediated systemic complement activation in these animals. These new findings indicate a global role of MBL during late diabetes-mediated vascular complications in various tissues.

## 1. Introduction

By 2014, the worldwide prevalence of diabetes mellitus was estimated to be 9% among adults above 18 years making it a serious global health issue [1]. Late vascular complications in diabetes include atherosclerosis, cardiovascular disease (CVD), retinopathy, neuropathy, and nephropathy [2]. Several studies have recognized both hyperglycemia and advanced glycation end products (AGEs) as major parts of the pathogenic pathway leading to diabetes-related micro- and macrovascular complications [3, 4]. Current therapeutic approaches are limited to controlling blood glucose levels and other risk factors, while no specific treatment has been marketed yet [5]. The complement system is part of the innate immune system and thus plays a vital role in the first line of immune defence, but it has also been suggested to play a direct pathogenic role in the development of late diabetes-mediated vascular complications [6–9]. The system may be divided into three distinct initiation pathways: the classical, the lectin, and the alternative. Activation of the lectin pathway is achieved through recognition of evolutionary conserved pathogen-associated molecular patterns (PAMPs) that are present on the surface of, for example, invading microorganisms [10]. The lectin pathway can be initiated by the collectins MBL, CL-K1, and CL-LK and all the ficolins [11, 12]. The binding of MBL leads to the activation of MBL-associated serine protease 1 (MASP-1), which is considered the initiator of the pathway. Activated MASP-1 cleaves the second MBL-associated serine protease 2 (MASP-2), which in turn cleaves the complement proteins C4 and C2 leading to the formation of the surface-bound C3 convertase. This leads to deposition of C3b onto adjacent pathogenic surfaces for enhanced recognition by immune cells and ultimately insertion of the membrane attack complex (MAC) [11]. Long-term uncontrolled hyperglycemia and the formation and accumulation AGEs have been proposed to result in a change in the glycation of cells, proteins, and endothelial surfaces [13–15]. Modification in the surface glycation pattern may in turn facilitate an altered immune response due to recognition of structurally altered self-surfaces and proteins. The involvement of the lectin pathway in the development of diabetic complications has attracted much attention following recently published studies indicating MBL as causally involved, and the lectin pathway may thus constitute a target for specific treatment [16–18].

We have recently shown increased binding of MBL in the kidney of diabetic mice by IVIS scanning and within the glomerulus by immunofluorescence in a type 1 diabetes mouse model [19, 20]. However, whether such MBL binding occurs throughout the body and whether this may lead to systemic complement activation remain unknown. The purpose of this study was therefore to further investigate the potential autoreactivity of MBL and possible complement activation in a type 1 diabetes mouse model.

## 2. Methods

### 2.1. Animals

We used 8-week-old male adult MBL/DKO C57BL/6J BomTac mice (Taconic, Ry, Denmark). Development of the MBL/DKO mouse strain has previously been described [21]. The different study groups are described below.

The animals were housed 6–8 per cage with free access to tap water and standard chow (Altromin #1324, Lage, Germany). Housing took place in a room with a 12-hour artificial light cycle (7 a.m. to 7 p.m.), a humidity of 55 ± 5%, and a constant temperature of 21 ± 1°C. All animals had an initial body weight of 10–24 g, and animals exhibiting signs of illness and more than 10% loss in body weight during the study were excluded. The study was terminated after 25 weeks. The study complied with the Danish regulations for care and use of laboratory animals.

### 2.2. Induction of Diabetes

Diabetes was induced by intraperitoneal (i.p.) injections with 50 mg streptozotocin (STZ) (Sigma-Aldrich Corp., St. Louis, MO, USA) per kg body weight during five consecutive days using the low-dose STZ protocol [22]. Prior to injection, all animals were fasted for 4 h with access to tap water only. On each day of injection, STZ was dissolved in freshly made room temperature (RT) sodium citrate dihydrate buffer (10 mmol/L, pH 4.5) (Thermo Fischer Scientific, MA, USA). Blood glucose and body weight were determined at baseline before induction. Responding animals exhibiting blood glucose levels above 15 mmol/L were considered diabetic. Poorly responding animals (<15 mmol/L) were excluded from the study.

### 2.3. Collection of Samples, Determination of Blood Glucose, and Albumin-to-Creatinine Ratio (AcR)

Blood samples were drawn by submandibular facial vein puncture every 14 days and collected in serum collection tubes (Sarstedt, Nürnbrecht, Germany). Samples were centrifuged at 1300*g* for 15 min at RT. Spot urine samples were also collected every 14 days. Blood glucose concentration was measured from tail vein blood using an Accu-Chek® Aviva Nano (Roche, Mannheim, Germany). Baseline samples were collected one week prior to STZ induction.

Urine creatinine was measured by isocratic UPLC as previously described in detail [19]. In brief, samples were initially diluted 20-fold in HPLC-grade acetonitrile (added with 0.5% HPLC glacial-grade acetic acid) followed by 10-fold in HPLC running solvent (5 mM sodium acetate ≥ 99.0% for HPLC, 4% HPLC glacial grade methanol, and 0.9% HPLC-grade AcN). Samples were stored at −20°C followed by centrifugation at 10,000*g* for 10 min at 4°C. Supernatants were transferred to new Eppendorf tubes followed by evaporation to dryness using a SpeedVac (Thermo Fisher Scientific, USA). The residual dried pellets were reconstituted with 25 *μ*L HPLC running solvent, and samples were run on a Zorbax SCX 300 column (Agilent Technologies, Wilmington, DE, USA) using the Acquity™ Ultra Performance (UPLC) System (Waters, Milford, MA, USA). All samples were run in duplicates. Data was analysed using Empower™ software. Corresponding urine albumin concentrations were analysed using a commercial ELISA quantification kit (Bethyl Laboratories Inc., TX, USA) according to the manufacturer's instructions. An albumin-to-creatinine ratio was subsequently calculated for each animal.

For determination of C3a plasma concentrations, blood was drawn from the submandibular facial vein pouch in potassium EDTA collection tubes (Sarstedt, Nürnbrecht, Germany). To inhibit ex vivo activation of the complement system, the samples were added with 1 *μ*L of 5 mg/mL nafamostat mesylate protease inhibitor (Futhan, FUT-175, BD Biosciences, Framingdale, NY, USA) per 100 *μ*L blood followed by mild shaking and storage on ice. Samples were centrifuged at 1300 *g* for 15 min at 4°C. Serum, EDTA plasma, and urine samples were stored at −80°C until analysed.

### 2.4. Recombinant MBL

Recombinant human MBL (rhMBL) (Enzon Pharmaceuticals, FL, USA) was labelled with the Alexa Fluor® 680 dye (AF680, cat# A200008) as follows: the AF680 dye was dissolved in dimethyl sulfoxide (DMSO) at 10 mg/mL and stored at −80°C. The rMBL was diluted in PBS to a concentration of 1 mg/mL followed by incubation in darkness with the AF680 dye at a molar ratio of 1 : 20 for 1 h at RT on a rocking table. This resulted in a theoretical degree of labelling (DOL) of approximately 15–21 AF680 dye per rMBL molecule. Any unbound dye was removed by dialysis against 2 L PBS (pH 7.4) for 2 × 2 h and 1 × overnight in SpectraPor dialysis tubes (Cole-Parmer, IL, USA) at RT. The tubing material was pre-blocked to avoid loss of protein by incubation with TBS/0.05% Tween (10 mM Tris-HCl, 140 mM NaCl, 15 mM sodium azide, and 0.05% (*v*/*v*) Tween 20, pH 7.4), followed by rinsing in ddH_2_O. Final protein concentration was determined using a NanoDrop1000 by correcting for the absorbance of the AF680 dye. The retained function of the labelled MBL was tested in a solid-phase ligand assay for MBL (see below).

### 2.5. Test of Labelled rMBL

The time-resolved immunofluorometric assay (TRIFMA) for quantification of MBL has previously been described in detail [23]. In brief, microtitre wells were coated with 10 *μ*g/mL mannan in carbonate coating buffer (15 mM Na_2_CO_3_, 35 mM NaHCO_3_, and 15 mM NaN_3_, pH 9.6). AF680-labelled rMBL and rMBL were incubated in either TBS/0.05% Tween/Ca^2+^ or TBS/0.05% Tween/10 mM EDTA on mannan-coated plates. In parallel, AF680-labelled rMBL and unlabelled rMBL were in the calcium-containing buffer including various concentrations of either mannose or galactose (0, 6.25, 12.5, 25, 50, and 100 mM) in TBS/0.05% Tween/Ca^2+^ for 1 h on rotation at RT. All samples were subsequently incubated on mannan-coated plates overnight at 4°C. The plates were washed thrice in TBS/0.05% Tween/Ca^2+^ followed by incubation for 2 h at RT with a biotin-labelled anti-MBL antibody. The wells were washed thrice in TBS/0.05% Tween/Ca^2+^ followed by incubation with Eu^3+^-labelled streptavidin for 1 h at RT, followed by another three washes and finally addition of 200 *μ*L enhancement solution in each well. Fluorescence was detected on a fluorometer (Victor^5^, Perkin Elmer, Waltham, MA, USA). Data is the given percentage of bound MBL with mean values obtained in the presence of galactose which were defined as 100% bound, and subsequent samples were calculated according to these. Data represents an average from two experiments.

### 2.6. IVIS Study

We initially randomized 8-week-old male MBL/DKO C57BL/6J mice (*n* = 29) into two groups: (1) diabetic (*n* = 16) and (2) nondiabetic (*n* = 13), and injected with low-dose STZ or buffer during five consecutive days (see Section 2.2). Development of diabetes was monitored by tail vein blood glucose determination every 14 days along with collection of blood and urine samples. Mice treated with STZ demonstrated average blood glucose levels above 15 mmol/L (17.7 mmol/L) during the first weeks after STZ induction (*n* = 12). Nonresponding animals and animals showing a sign of illness or malcontent were sacrificed and excluded (*n* = 5). After additional 25 weeks of diabetes, the mice were further subdivided into four groups: (1) diabetic + MBL (*n* = 6), (2) diabetic + PBS (*n* = 6), (3) nondiabetic + MBL (*n* = 6), and (4) nondiabetic + PBS (*n* = 6). Animals in groups 1 and 3 were administered with 800 pmol/kg AF680 rMBL by i.v. injection. Animals in groups 2 and 4 were injected i.v. with PBS.

After another 24 hours, the mice were anaesthetized by i.p. injections with a mixture of 0.5 mg ketamine (Ketaminol® Vet, Intervet, Skovlunde, Denmark) and 0.2 mg xylazine (Narcoxyl® Vet, Intervet, Skovlunde, Denmark) per gram body weight. The following organs were dissected under full anaesthesia from each animal followed by sacrifice by cervical dislocation: (1) heart, (2) liver, (3) brain, (4) spleen, (5) lungs, (6) part of the pancreas, (7) part of the small intestine, and (8) part of the colon. All organs were rinsed thrice in PBS and weighed prior to ex vivo scanning. The right lobe of the liver and the superior left lobe of the lungs from each mouse were scanned. Similar dissected parts of the small intestine, large intestine, and pancreas were weighed to ensure comparable fluorescence signal intensity in the IVIS imager. The following excitation and emission filter settings were used: 675/720, 675/740, 675/760, 675/780, 660/605, 680/605, 700/605, 720/605, 740/605, 760/605, and 780/605.

All acquired data were processed using Living Image® 4.5 (Caliper Life Science). Quality control was carried out using only raw data with counts above 600 and below 6 × 10^4^ in the specific image color scale. The calibrated unit radiant efficiency was used to obtain a quantitative output, as it accounts for the nonuniformity of the light projection on the stage and the associated excitation light. This will normalize for the excitation lighting intensity per square area of the field of view, resulting in an average radiant efficiency unit given as (photons/sec/cm^2^/sr)/(*μ*W/cm^2^).

### 2.7. Complement Activation

A second group of age-matched male MBL/DKO C57BL/6J mice (*n* = 26) was randomized into two groups: diabetic (*n* = 14) and nondiabetic (*n* = 12). Diabetes was induced as previously described (see above). Animals exhibiting poor metabolic response to STZ treatment and showing a sign of illness or malcontent were sacrificed and excluded (*n* = 4). After additional 25 weeks of hyperglycemia, the groups were further divided into the following: (1) diabetic + MBL (*n* = 6), (2) diabetic + PBS (*n* = 5), (3) nondiabetic + MBL treatment (*n* = 6), and (4) nondiabetic + PBS (*n* = 5). Baseline blood samples were taken at 0 h followed by i.v. tail vein injection with either 800 pmol/kg body weight AF680-labelled MBL or equal volume PBS pH 7.4. End-blood samples were taken by retro-orbital puncture after 24 h, followed by sacrifice by cervical dislocation.

Complement activation was accessed by measuring plasma concentrations of C3a in a sandwich-based TRIFMA assay modified from Malik et al. [24]. Although C3 does contain the peptide sequence of C3a, the assay does not cross-react with C3 as it detects neoepitopes generated by cleavage of C3. In brief, microtitre plates (Nunc Maxisorp) were coated with 100 ng purified rat anti-mouse C3a (BD Pharmingen, 558250) per 50 *μ*L BBS (100 mM H_3_BO_3_, 25 mM Na_2_B_4_O_7_, and 75 mM NaCl, pH 8.3) and incubated at 4°C overnight. Residual binding sites were blocked with PBS (137 mM NaCl, 2.7 mM KCl, 1.5 mM KH_2_PO_4_, and 8.1 mM Na_2_HPO_4_, pH 7.4) containing 1% (*v*/*v*) Tween 20 (Tw) for 1 h at room temperature (RT). The plates were washed three times in C3a washing buffer (PBS added with 0.05% (*v*/*v*) Tw) and kept on ice for sample application. The EDTA-plasma samples were analysed in three different dilutions. They were initially thawed on ice and diluted (1 : 40, 1 : 80, and 1 : 160) in cold C3a sample buffer (PBS added with 2% (*v*/*v*) BSA, 0.1% (*v*/*v*) Tw, and 0.02% (*v*/*v*) NaN_3_) and applied to the plate followed by incubation at 4°C overnight. Purified mouse C3a protein (BD Pharmingen, 558618) containing 100 ng C3a/mL was diluted 1.5-fold 10 times in C3a sample buffer to create a standard curve. All samples were added in duplicates along with a buffer-only control. The plates were washed five times in cold C3a washing buffer. Bound C3a was detected by adding 50 *μ*L biotin-labelled rat anti-mouse C3a (BD Pharmingen, 558251) at 1 *μ*g/mL dilution in C3a sample buffer per well and incubated for 5 h at 4°C. The plates were washed five times in cold C3a washing buffer. Wells were added with Eu^3+^-labelled streptavidin diluted to 1 : 1000 in C3a washing buffer added with 25 *μ*M EDTA and incubated for 1 h at RT. The wells were washed five times in cold C3a washing buffer and read as described above.

### 2.8. Statistics

**Figure 1:**
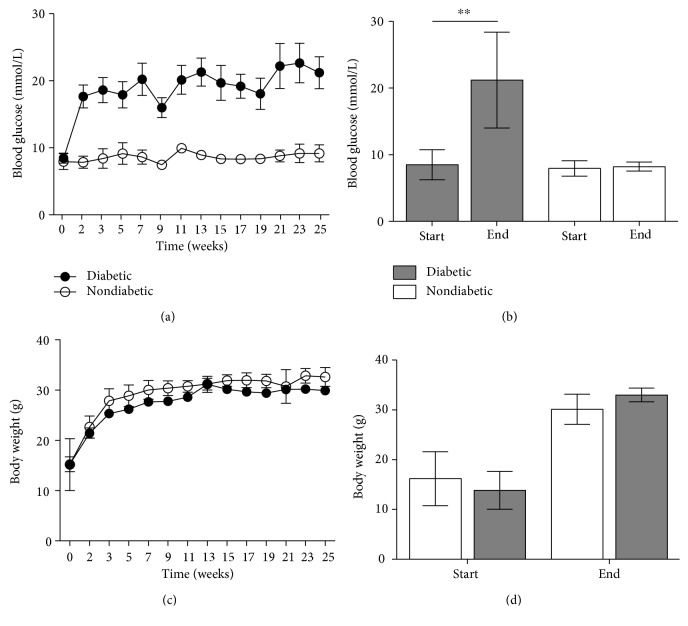
Measurement of blood glucose and weight in diabetic and control mice. (a) Blood glucose measurement of diabetic and nondiabetic mice, (b) change in blood glucose from start to end of the study for diabetic (start: *n* = 16, end: *n* = 12) and nondiabetic (start: *n* = 13, end: *n* = 12) mice. (c) difference in body weight during the study, (d) difference in body weight from start to end of the study for diabetic (start: *n* = 16, end: *n* = 12) and nondiabetic (start: *n* = 13, end: *n* = 12) mice. Student's *t*-test was used to test the difference between groups, which was indicated in the figure when significant. ^∗∗^*p* < 0.01. Data is given in mean with standard deviations (SDs).

**Figure 2:**
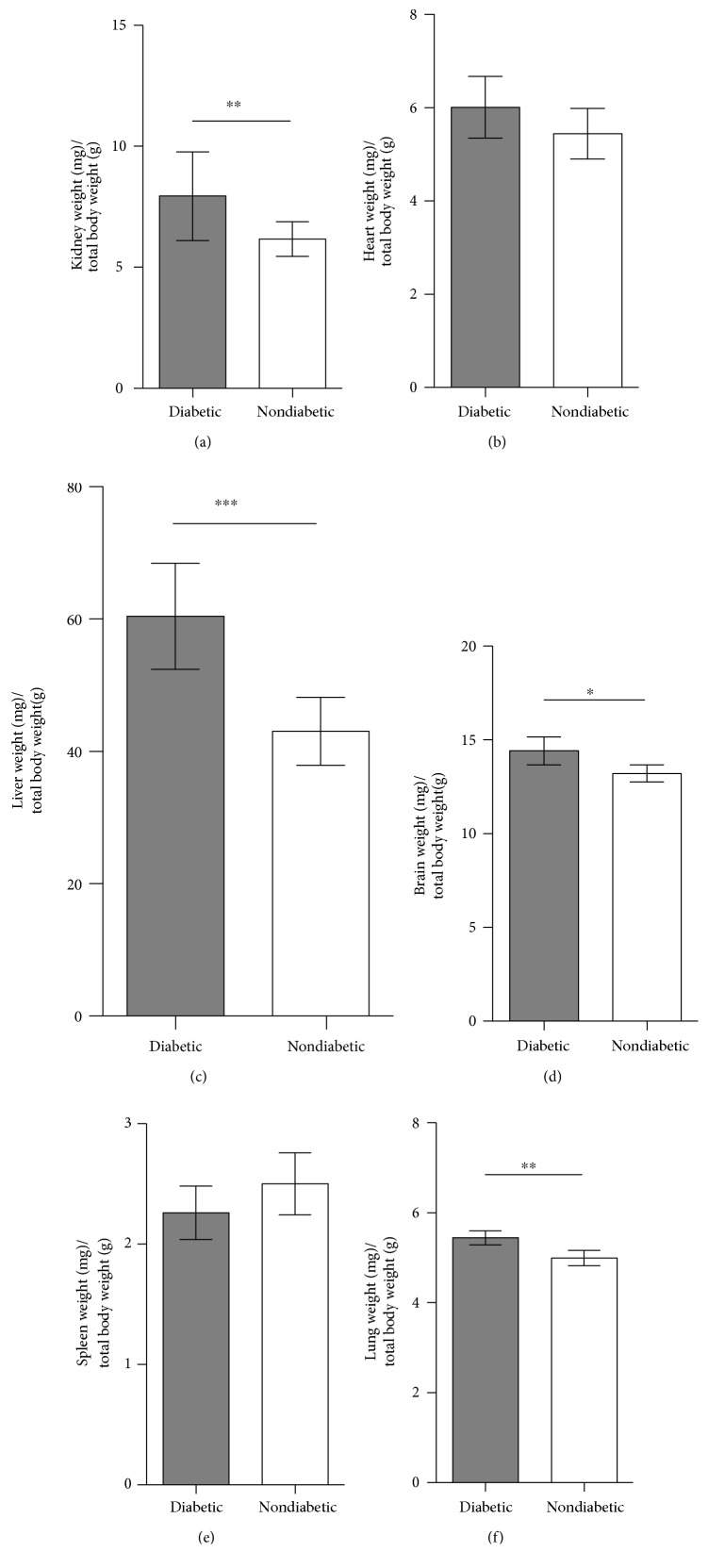
Organ-to-body weight ratio of diabetic and control mice. (a) Weight ratio measurements at the end of the study of diabetic (*n* = 12) and nondiabetic (*n* = 12) mice in the (a) kidney, (b) heart, (c) liver, (d) brain, (e) spleen, and (f) lung. Student's *t*-test was used to test the difference between groups, which was indicated in the figure when significant. ^∗^*p* < 0.05, ^∗∗^*p* < 0.01, ^∗∗∗^*p* < 0.001. Data is given in mean with standard deviations (SDs).

**Figure 3:**
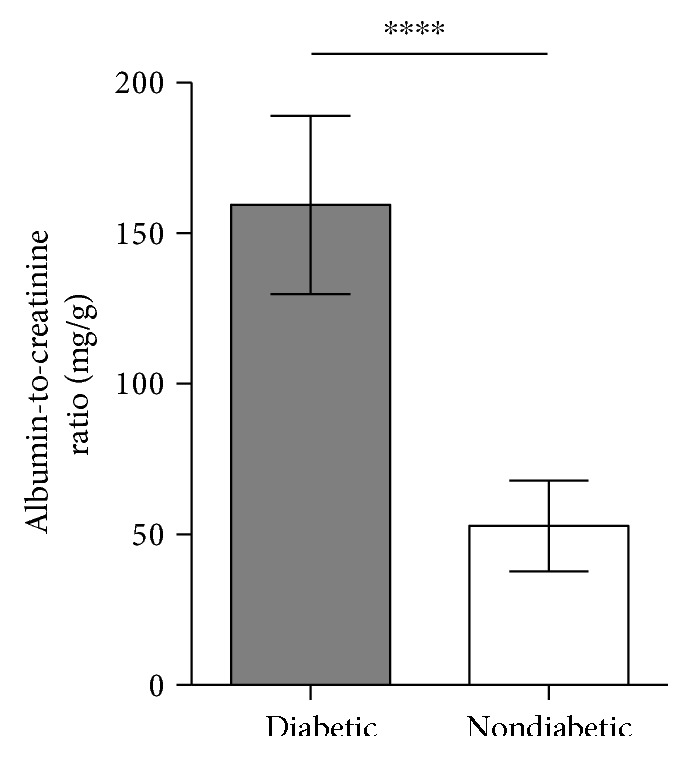
Albumin-to-creatinine ratio (AcR) in diabetic and nondiabetic mice. AcR measurements of diabetic (*n* = 12) and nondiabetic (*n* = 12) mice at the end of the study. Student's *t*-test was used to test the difference between groups. ^∗∗∗∗^*p* < 0.0001. Data is given in mean with standard deviations (SDs).

Student's *t*-test was used to compare start and end values between the diabetic and control groups in Figures 1, 2, and 3. Two-way ANOVA was used to test the interaction between diabetes status (nondiabetic/diabetic) and MBL treatment (MBL/PBS), (*P*_int_), that is, does diabetes modify the possible autorecognition of MBL. Furthermore, complement activation as a consequence of diabetes was evaluated by Student's paired *t*-test and by comparing delta values (0 h–24 h) between the diabetic and nondiabetic group in response to treatment with MBL or PBS. Unless otherwise stated, all data is given in mean with standard deviations (SDs), with *N* indicating the number of observations/mice per group. *p*values < 0.05 were considered significant. All statistical tests for normality were made using Stata 13 (StataCorp LP, College Station, TX, USA), while *t*-tests and two-way ANOVA were made using GraphPad Prism 5.0 (GraphPad Software Inc., La Jolla, CA, USA).

## 3. Results

### 3.1. Animal Characteristics—Blood Glucose

Blood glucose levels were identical in the diabetic group and nondiabetic group at the start of the study (*p* = 0.51) (Figure 1(a)). Blood glucose in the diabetic group was elevated during the study compared to that of the nondiabetic group. Measurements at the end of the study were 21.2 mmol/L in the diabetic group compared to 8.2 mmol/L in the nondiabetic group (*p* = 0.0004).

### 3.2. Animal Characteristics—Body Weight

The total weight gain did not at any point diverge between the diabetic group and the nondiabetic group during the 25 weeks, and all mice exhibited solid weight gain. The body weight in the two groups did not diverge during the study (Figure 1(c)). Measurements at the end of the study were 30.1 g in the diabetic group compared to 33.0 g in the nondiabetic group (*p* = 0.08) (Figure 1(d)).

### 3.3. Organ-to-Body Weight Ratio and Albumin-to-Creatinine Ratio (AcR)

The overall kidney-to-body weight ratio increased by 24.6% in the diabetic group compared to the nondiabetic group (*p* = 0.008) (Figure 2(a)). We furthermore observed a diabetes-induced weight change of several organs. The liver of diabetic mice showed a 31.9% increase in organ-to-weight ratio compared to that of nondiabetic mice (*p* = 0.0002) (Figure 2(c)). Surprisingly, the diabetic group also exhibited a 13.7% increased brain-to-body weight ratio compared to nondiabetic mice (*p* = 0.02). Moreover, we observe an average increase of 10% in lung-to-body weight in diabetic animals as compared to control mice (*p* = 0.004) (Figure 2(f)). There was no effect of diabetes on the heart-to-body weight ratio and the spleen-to-body weight ratio (*p* = 0.10 and *p* = 0.07, resp.) (Figures 2(b) and 2(e)).

The albumin-to-creatinine ratio was lower in the diabetic MBL/KO mice compared to previously reported levels in WT mice (submitted manuscript) but significantly higher compared to that in the nondiabetic group, 164.2 mg/g (CI: 138.2–180.7) versus 54.9 mg/g (CI: 42.6–63.0) (*p* < 0.0001) (Figure 3).

### 3.4. Functional Test of Labelled rMBL

**Figure 4:**
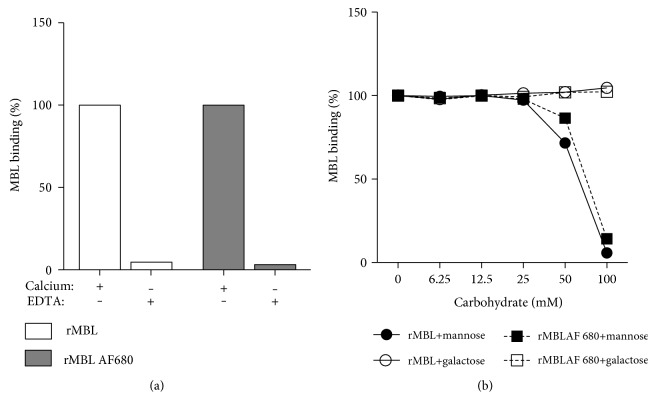
Test for preserved functionality of labelled rMBL. (a) Unlabelled and labelled rMBL were incubated on mannan-coated plates in calcium- or EDTA-containing buffers. Values measured in calcium buffer were defined as 100%, and subsequent samples were calculated according to these. (b) Unlabelled and labelled rMBL were incubated on mannan-coated plates in the presence of either mannose or galactose. Values acquired in the presence of galactose were defined as 100%, and subsequent samples were calculated according to these. Representative of two individual experiments.

The labelled rMBL was tested for preserved functionality and compared to the unlabelled rMBL stock. Both the calcium-dependent binding activity and the sugar specificity were evaluated by incubation with either calcium- or EDTA-containing buffer and by incubation with various concentrations of mannose or galactose. There was no noticeable difference between labelled and unlabelled rMBL in the calcium-dependent binding to ligands, and further incubation with carbohydrates revealed a dose-dependent inhibition of both rMBL preparations with mannose and not galactose (Figures 4(a) and 4(b)).

### 3.5. IVIS Study

**Figure 5:**
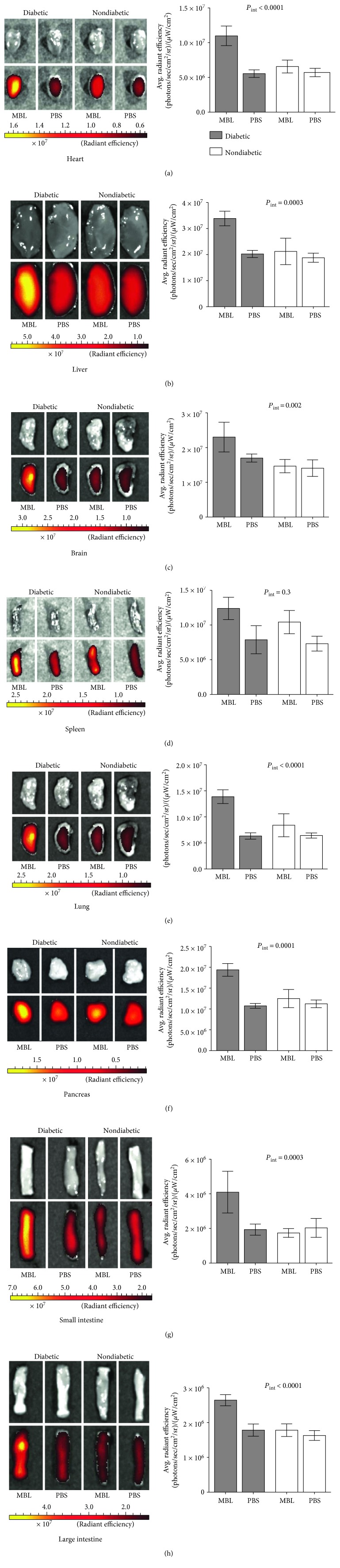
Ex vivo imaging and region of interest (ROI) quantification of selected organs from diabetic and nondiabetic mice injected with either AF680-labelled rMBL or PBS. (a) Heart, (b) liver (right lobe), (c) brain, (d) spleen, (e) lung (superior left lobe), (f) part of the pancreas, (g) part of the small intestines, (h) and part of the large intestines. Left side, top: CCD photo, bottom: fluorescent image. Scale bar is given in radiant efficiency (photons/sec/cm^2^/sr)/(*μ*W/cm^2^). Two-way ANOVA was used to test the difference between groups (*P*_int_). Data is expressed in mean with standard deviations (SDs). Images are presented as CCD camera photo and fluorescence images. The experiment was repeated twice, and the data is based on 6 individual mice in each group (*n* = 24 total). Data was obtained with similar results.

As described, we have previously observed that MBL accumulates in the kidney in a similar animal model in response to diabetes. In this study, we further investigated the possible autorecognition by MBL in various tissues in response to long-term uncontrolled hyperglycemia. Using two-way ANOVA analysis, we observed significantly higher MBL accumulation in the heart, liver, brain, lung, pancreas, and intestines of diabetic mice compared to that in those of nondiabetic mice. No such accumulation was seen in the spleen. Furthermore, we found no significant MBL accumulation in the nondiabetic groups when comparing to PBS-injected animals (Figures 5(a), 5(b), 5(c), 5(d), 5(e), 5(f), 5(g), and 5(h)).

### 3.6. Complement Activation in Mice

**Figure 6:**
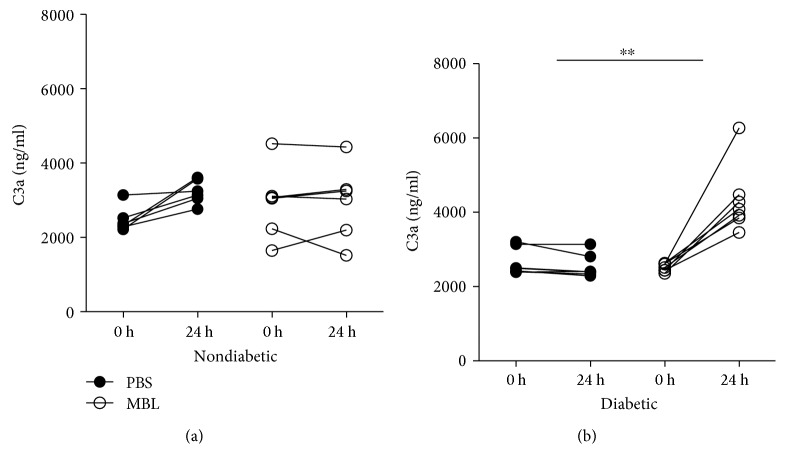
Circulating C3a as a measurement of complement activation. The difference in change of C3a plasma levels from 0 h to 24 h between the nondiabetic (a) and the diabetic (b) groups was tested by Student's *t*-test on delta values and indicated in the figure when significant. ^∗∗^*p* < 0.01. Data is given in mean with standard deviations (SDs).

In order to evaluate whether the observed self-recognition of MBL would lead to complement activation, we measured plasma levels of C3a at baseline just prior to injection with MBL and again after 24 hours. When comparing the change in C3a concentration during 24 h between the two treatment groups (delta values in the PBS group versus delta values in the MBL group), we found no significant difference between treatment with MBL and that with PBS in the nondiabetic group (*p* = 0.16), but we observed a significant difference in C3a concentration during the study between the PBS- and MBL-treated diabetic mice, thus indicating an elevated systemic complement activity in diabetic MBL-knockout mice injected with MBL (*p* = 0.003) (Figures 6(a) and 6(b)).

## 4. Discussion

The present study further supports the hypothesis that autoreactivity of MBL plays a role during the development of late vascular complications in diabetes. Besides increased binding in the diabetic kidney, we here demonstrate that MBL accumulates in several other sites. We furthermore found an increased systemic complement activation by measurement of plasma C3a levels, thus demonstrating downstream complement activity. It has previously been shown that high MBL serum levels are associated with increased risk of progression to microalbuminuria in patients with type 1 diabetes [6, 16]. Later studies have furthermore demonstrated a predictive role for high MBL serum levels for the development of micro- and macroalbuminuria [25]. In recent studies, we demonstrated that MBL indeed accumulates in the kidneys of diabetic mice with late vascular complications, thus strengthening the hypothesis of an autoreactive role of MBL in diabetic nephropathy [19, 20]. Our present findings further link the complement system to the pathophysiology of complications in type 1 diabetes.

The lectin pathway of the complement system may be activated by MBL upon recognition and binding to specific carbohydrate structures found on invading microorganisms [10]. This initiates a local inflammatory response with deposition of C3b fragments for enhanced phagocytosis by, for example, activated macrophages, release of various different inflammatory cytokines and chemokines (C3a and C5a), and ultimately cell lysis by the formation of MAC [11]. Besides recognizing evolutionary conserved patterns on, for example, certain bacteria and viruses, the complement PRMs may also recognize disease-mediated altered self-surfaces [26, 27]. Cellular stress, hypoxia, and apoptosis may result in change of surface structure. MBL has been suggested to play a role in several other autoimmune diseases besides type 1 diabetes [28–30]. Diabetes mellitus has been associated with increased oxidative stress and reduced NO signalling, thus resulting in inflammation, remodelling of the arterial vessels, and malfunction of vascular endothelium [31, 32]. The late complement activation components C5b-9 (MAC) have previously been shown to play an important role in the loss of NO-mediated relaxation of arterial vessels [33]. More recent studies have demonstrated that the NO-mediated relaxation of vessels is partially MBL-dependent [34]. In vitro studies have suggested that MBL is able to bind to hyperglycemia-induced neoepitopes of the Amadori type and activate complement [35, 36]. By two supplementary designed studies, we have recently found MBL to accumulate in the kidney in an in vivo IVIS study and in the glomerulus by immunofluorescence [19, 20]. As vascular tissue and organs throughout the body are exposed to hyperglycemia in diabetes, autoattack of MBL could potentially occur in several sites. We therefore tested if long-term diabetes would modify the autorecognition capability of MBL.

The effect of diabetes resulted in a significantly higher accumulation of MBL in the heart of diabetic mice, suggesting an altered tissue glycation pattern. The relative heart-to-body weight between diabetic and nondiabetic mice was however not significantly different although a slight tendency of increased heart volume was found in the diabetic mice. This is in good agreement with a previous study, which demonstrated that MBL-null mice were protected from myocardial injury after hyperglycemia and that they exhibited conditions similar to those of insulin-treated hyperglycemia mice [37]. A later study involving myocardial histochemistry furthermore showed that hyperglycemic MBL-null mice exhibited a significantly lower degree of altered tissue morphology compared to wild-type mice [34]. These studies together with our present work suggest a strong link between MBL and myocardial injury in response to uncontrolled hyperglycemia.

Liver disease has also been associated with type 1 diabetes. A longitudinal cohort study demonstrated that a significant part of patients with type 1 diabetes had histological evidence of both tissue fibrosis and cirrhosis [38]. Glycogen accumulation in liver tissue is observed in 80% of diabetic patients possibly due to enhanced gluconeogenesis and defective activation of glycogen synthase. Excessive cytoplasmic glycogen buildup may lead to hepatomegaly along with liver enzyme abnormalities. Also, hepatic fat accumulation in the form of triglycerides is well known to be associated with uncontrolled hyperglycemia in type 1 diabetes, which may further lead to steatosis and progression to micro- and macrovascular fibrosis [39]. This is in good agreement with the observed higher relative liver-to-body weight ratio of diabetic mice compared to that of nondiabetic mice. In agreement, we observed a significantly higher deposition of MBL in the diabetic liver compared to that of the nondiabetic. The excessive amount of glucose circulating during hyperglycemia leads to formation of AGEs through the Maillard reaction [40]. Glucose may spontaneously react with free amines forming Schiff bases. These are short-lived and will further rearrange Amadori products, such as fructosamines and Hb1Ac, which then finally rearrange to produce AGEs. A recent study has established a possible direct link between hyperglycemia and complement activation by demonstrating that MBL may bind to the pre-AGE product fructosamines [35]. Fructosamine and mannose share the same orientation of the 3′and 4′OH group in the equatorial plan necessary for MBL recognition. The final step from the Amadori product to AGEs occurs over weeks and months. This means that fructosamine is accessible for recognition and binding by MBL for a long period of time. AGEs are formed by both oxidative and nonoxidative reactions of sugars followed by adduction to, for example, cell membrane, proteins, and lipids [41]. Oxidative stress leads to alteration in the cell surface glycosylation pattern, thus mediating a possible ligand for MBL binding [26]. Both the kidney and the liver play an important role in the clearance and catabolism of AGEs, and it is plausible that MBL may bind to liver-derived pre-AGE structures, such as fructosamines bound to host proteins, thereby mediating local inflammation and fibrosis.

Several studies concerning late complications associated with type 1 diabetes and uncontrolled hyperglycemia have implicated gross morphological changes in brain structure [42–44]. Patients with chronic hyperglycemia appear to be at particular risk of developmental changes in the brain [45]. In agreement, we observed significantly higher accumulation of MBL in the brain of diabetic mice compared to that of control mice. We found a significant increase in the brain-to-body weight ratio in the diabetic group compared to that in the nondiabetic group. The neurocognitive changes in response to microvascular disease associated with type 1 diabetes have been suggested to be linked to reduction in both white and gray matter volume densities [42, 44].

No effect of diabetes on MBL self-recognition was found in the spleen. Thus, no difference in the accumulation of MBL between of diabetic and of nondiabetic mice was observed. This result was somewhat unexpected since the spleen has a main role in both immunity and removal of red blood cells. There was similarly no difference in the spleen-to-body weight ratio between diabetic and control mice.

Late complications of type 1 diabetes have furthermore been shown to modify the function and structures of the lungs causing microangiopathy [46]. Restrictive lung disease in diabetes may develop from chronic low-grade tissue inflammation, microangiopathy, and accumulation of nonenzymatic pre-AGEs and AGEs [47–50]. We observed a significantly higher signal of MBL accumulated in the diabetic lung. Also, the weight of the diabetic lungs was significantly elevated suggesting a diabetes-mediated tissue alteration.

We moreover investigated if the potentially altered host surfaces in combination with MBL self-recognition would lead to complement activation by the lectin pathway. Indeed, after injection with MBL, we observe a significant increase in plasma C3a fragment in diabetic mice compared to that in nondiabetic control mice. This strongly indicates an elevated complement system response. Our present results indicate that MBL binds to various tissues in mice with long-term diabetes, and this binding is associated with a significant increase in complement activation. This could possibly be an important early step in the development of diabetic complications.

In conclusion, we observed a strong effect of diabetes on the binding of MBL to targets in several tissues. Diabetic animals injected with MBL demonstrated elevated C3a blood levels compared to diabetic animals injected with PBS. Our present results suggest that the effect of diabetes on MBL self-recognition results in an increased complement activation and thus an increased inflammatory state.
